# Supplementary material for: Comparative venomics suggests an evolutionary adaption of spider venom from predation to defense
Source: Commun Biol. 2025 Oct 23;8:1496. doi: 10.1038/s42003-025-09015-6 (PMC12550024; doi:10.1038/s42003-025-09015-6)
Supplement: Supplementary file 2 — Supplementary Information [file 42003_2025_9015_MOESM2_ESM.pdf]

Supplementary Tables S1. Sampling and sequencing data for spiders used in comparative venomics. Subtable A) lists all collected spiders, obtained sample type and sample size alongside the collection locality. Subtable B) summarizes sequencing chemistry, methods and quality metrics.

A)

**Taxon**	**Sample Type**	**Locality**	**Number**
Cheiracanthium punctorium	Venom, Venom glands	Görlitz, Germany	8 male, 12 female
Thanatus vulgaris	Venom glands	Eiting, Germany (invasive)	10 male, 10 female
Meta menardi	Venom glands	Saarland, Germany	10 male, 10 female
Larinioides sclopetarius	Venom glands	Gießen, Germany	6 male, 8 female
Psalmopoeus reduncus	Venom glands	Costa Rica, Pet Trade	1 female

B)

**Taxon:**	***Cheiracanthium punctorium***	***Larinioides sclopetarius***	***Meta menardi***	***Thanatus vulgaris***	***Psalmopoeus reduncus***
sequencing kit	SMARTer Universal Low Input RNA for Sequencing,<br>TruSeq RNA Library Preparation v2 Kit	SMART-Seq v4 Ultra Low Input RNA Kit,<br>TruSeq RNA Sample Prep Kit v2, TruSeq Stranded mRNA LT Sample Prep Kit	SMART-Seq v4 Ultra Low Input RNA for Sequencing,<br>TruSeq RNA Library Preparation v2 Kit	SMART-Seq v4 Ultra Low Input RNA for Sequencing,<br>TruSeq RNA Library Preparation v2 Kit	TruSeq Stranded mRNA Library Prep Kit
strand specific	-	X	-	-	X
Illumina platform	MiSeq	MiSeq	NextSeq	NextSeq	NextSeq/NovaSeq
read length	151	151	151	151	151
minimum length trimming	30	30	30	30	25
stats					
raw reads	21,902,394	27,304,547	18,953,819	17,223,809	43,069,379
filtered reads	21,792,272	2,720,989,200	12,189,239	10,549,401	41,806,291
filtered reads [%]	99.5	99.7	64.3	61.2	97.1
contigs	1,370,688	292,908	195,124	258,077	484,816
overall alignment rate [%]	91.93	98.88	97.34	91.63	97.80
unique ORFs	5,520,137	1,576,422	1,177,741	1,631,215	3,224,650
unique ORFs with InterPro	65,304	35,790	18,487	14,054	-
unique ORFs with InterPro, SP	765	1,653	859	767	-
unique ORFs with SP	-	-	-	-	7,250
unique ORFs with SP, InterPro	-	-	-	-	1,266
