# Supplementary material for: Comparative venomics suggests an evolutionary adaption of spider venom from predation to defense
Source: Commun Biol. 2025 Oct 23;8:1496. doi: 10.1038/s42003-025-09015-6 (PMC12550024; doi:10.1038/s42003-025-09015-6)
Supplement: Supplementary file 3 — Description of Additional Supplementary Files [file 42003_2025_9015_MOESM3_ESM.pdf]

Description of Additional Supplementary Files

File name: Supplementary Data 1
Description: Venomics data from the venom of C. punctorium, incl. source data to generate figure 1 and figure 2.

File name: Supplementary Data 2
Description: Venomics data from the venom gland of M. menardi, incl. source data to generate figure 2.

File name: Supplementary Data 3
Description: Venomics data from the venom gland of P. reduncus, incl. source data to generate figure 2.

File name: Supplementary Data 4
Description: Venomics data from the venom gland of L. sclopetarius, incl. source data to generate figure 2.

File name: Supplementary Data 5
Description: Venomics data from the venom gland of T. vulgaris, incl. source data to generate figure 2.

File name: Supplementary Data 6
Description: Source data used to create the phylogenetic tree displayed in figure 4.
