# Supplementary material for: Comparative venomics suggests an evolutionary adaption of spider venom from predation to defense
Source: Commun Biol. 2025 Oct 23;8:1496. doi: 10.1038/s42003-025-09015-6 (PMC12550024; doi:10.1038/s42003-025-09015-6)
Supplement: Supplementary file 10 — Reporting Summary [file 42003_2025_9015_MOESM10_ESM.pdf]

nature portfolio

Corresponding author(s): Tim Lüddecke

Last updated by author(s): 21.07.2025

Reporting Summary

Nature Portfolio wishes to improve the reproducibility of the work that we publish. This form provides structure for consistency and transparency in reporting. For further information on Nature Portfolio policies, see our Editorial Policies and the Editorial Policy Checklist.

Statistics

For all statistical analyses, confirm that the following items are present in the figure legend, table legend, main text, or Methods section.

n/a	Confirmed	
☒	☐	The exact sample size (*n*) for each experimental group/condition, given as a discrete number and unit of measurement
☒	☐	A statement on whether measurements were taken from distinct samples or whether the same sample was measured repeatedly
☒	☐	The statistical test(s) used AND whether they are one- or two-sided<br>*Only common tests should be described solely by name; describe more complex techniques in the Methods section.*
☒	☐	A description of all covariates tested
☒	☐	A description of any assumptions or corrections, such as tests of normality and adjustment for multiple comparisons
☒	☐	A full description of the statistical parameters including central tendency (e.g. means) or other basic estimates (e.g. regression coefficient) AND variation (e.g. standard deviation) or associated estimates of uncertainty (e.g. confidence intervals)
☒	☐	For null hypothesis testing, the test statistic (e.g. *F*, *t*, *r*) with confidence intervals, effect sizes, degrees of freedom and *P* value noted<br>*Give P values as exact values whenever suitable.*
☒	☐	For Bayesian analysis, information on the choice of priors and Markov chain Monte Carlo settings
☒	☐	For hierarchical and complex designs, identification of the appropriate level for tests and full reporting of outcomes
☒	☐	Estimates of effect sizes (e.g. Cohen's *d*, Pearson's *r*), indicating how they were calculated

Our web collection on statistics for biologists contains articles on many of the points above.

Software and code

Policy information about availability of computer code

Data collection

Not applicble

Data analysis

All software used is publicly available, either as commercial product or open source data - no self-made scripts and program were used. Specifically, we used the below programs for analyzing the various types of data. For software where source code is publicly available, the link is given in brackets. Further details are provided in the material and method section of the manuscript.

Transcriptomics:
- FastQC v0.11.9/0.12.1 (www.bioinformatics.babraham.ac.uk); quality control of reads
- cutadapt v4.2/4.9; read trimming
- Rcorrector v1.0.5/1.0.7; trimmed read correction
- Trinity v2.13.2/2.15.1 and rnaSPAdes v3.15.5; transcriptome assembly
- CD-HIT-EST v4.8.1 and fastanrdb v2.4.0 (github.com/nathanweeks/exonerate); merging of assemblies
- HISAT2 v2.2.1; read re-mapping
- StringTie v2.2.1/2.2.2; TPM calculation
- samtools v1.16.1/1.20; SAM/BAM file conversion
- TransDecoder v5.5.0/5.7.1 (github.com/TransDecoder/TransDecoder); ORF prediction

Proteomics:
- Xcalibur v4.3.73.11 and Proteome Discoverer v2.4.0.305; mass spectrometry data acquisition and analysis
- Mascot v2.6.2; proten identification in bottom-up proteomics

In silico analysis of sequences and annotation:
- InterProScan v5.61-93.0/5.69-101.0 and DIAMOND v2.0.15/2.1.9 (also features from BioPython v1.81/1.83); annotation
- SignalP v6.0g/h; prediction of signal peptides
- Geneious v10.2.6; comparative alignments using ClustalW (integrated)
- Alphafold 3 (integrated in the Galaxy platform); protein structure models
- ChimeraX; protein model visualization
- IQ-TREE and iTOL webserver; phylogenetic analysis and visualization

For manuscripts utilizing custom algorithms or software that are central to the research but not yet described in published literature, software must be made available to editors and reviewers. We strongly encourage code deposition in a community repository (e.g. GitHub). See the Nature Portfolio guidelines for submitting code & software for further information.

Data

Policy information about availability of data

All manuscripts must include a data availability statement. This statement should provide the following information, where applicable:

- Accession codes, unique identifiers, or web links for publicly available datasets
- A description of any restrictions on data availability
- For clinical datasets or third party data, please ensure that the statement adheres to our policy

Raw proteomic data are available via ProteomeXchange with identifier PXD061529. Raw transcriptomic data have been uploaded to the European Nucleotide Archive (ENA) (Study PRJEB86488). The following data are available as supplements: Information on sampled spiders and their sequencing (Supplementary S1), proteotranscriptomic analysis of C. punctorium (Supplementary S2), Transcriptomic analysis of M. menardi (Supplementary S3), P. reduncus (Supplementary S4), L. sclopetarius (Supplementary S5), T. vulgaris (Supplementary S6), as well as models generated in Alphafold 3 (Supplementary S7), and the retrieved optimal phylogenetic tree (Supplementary S8).

Research involving human participants, their data, or biological material

Policy information about studies with human participants or human data. See also policy information about sex, gender (identity/presentation), and sexual orientation and race, ethnicity and racism.

Reporting on sex and gender	Not applicable.
Reporting on race, ethnicity, or other socially relevant groupings	Not applicable.
Population characteristics	Not applicable.
Recruitment	Not applicable.
Ethics oversight	Not applicable.

Note that full information on the approval of the study protocol must also be provided in the manuscript.

Field-specific reporting

Please select the one below that is the best fit for your research. If you are not sure, read the appropriate sections before making your selection.

☐ Life sciences ☐ Behavioural & social sciences ☒ Ecological, evolutionary & environmental sciences

For a reference copy of the document with all sections, see nature.com/documents/nr-reporting-summary-flat.pdf

Ecological, evolutionary & environmental sciences study design

All studies must disclose on these points even when the disclosure is negative.

Study description	Our work investigates the venom profile of the Cheiracanthium punctorium spider and several other species from closely related and more distant spider lineages via proteomics, transcriptomics, or a combination thereof. The gathered protein sequences are further used to investigate the evolutionary history, structural properties and biological function in context of natural history of the investigated species.
Research sample	The samples are dissected venom glands, crude venom, or a combination thereof from spiders taken from the wild. The samples have been taken following the established best practices in modern venomics, including physiological state normalization by keeping them under the same controlled conditions for extended times and meticolous timing of venom collection.
Sampling strategy	We collected the specimen from the wild or via commercial sourcing and for sample sizes oriented to values commonly used in our

Sampling strategy	field.
Data collection	The data was collected by the corresponding author who collected and processed the spiders.
Timing and spatial scale	The collection and processing of the spiders was carried out in autumn 2023 (01.08.2023-31.10.2023) in Germany.
Data exclusions	No data was excluded.
Reproducibility	For each of the venomic analyses, we have handed the data to various coauthors who independently analyzed it. The results of this internal benchmarking where compared to the data presented in our study to verify it and to test for reproducibility. All raw data are uploaded to public databases and allow for independent reproduction.
Randomization	We have randomly collected spiders from their natural environment and have pooled the samples following the best practices proposed for our field.
Blinding	Not applicable.

Did the study involve field work? ☒ Yes ☐ No

Field work, collection and transport

Field conditions	Fieldwork was carried out in autumn 2023 (01.08.2023-31.10.2023) in Germany. We selected warm (>25°C) days without rainfall and collected between 12 and 6pm. Fieldwork conditions did not have effect on the gathered data s collected spiders were kept under controlled conditions (room temperature with a ~12-h photoperiod for at least 1 week in appropriately-sized plastic enclosures (P. reduncus = 30x30x30 cm, all others 10x10x10 cm) containing potting soil as a substrate) for at least one week to secure physiological normalization in accordance to the best practices in our field.
Location	All visited sites are located in germany, we collected at Gießen, Neunkirchen, and Eitting.
Access & import/export	No import of samples was needed, no permits for collection were needed (none of the spiders is protected, no protected areas were visited).
Disturbance	None.

Reporting for specific materials, systems and methods

We require information from authors about some types of materials, experimental systems and methods used in many studies. Here, indicate whether each material, system or method listed is relevant to your study. If you are not sure if a list item applies to your research, read the appropriate section before selecting a response.

Materials & experimental systems

n/a	Involved in the study
☒	☐ Antibodies
☒	☐ Eukaryotic cell lines
☒	☐ Palaeontology and archaeology
☐	☒ Animals and other organisms
☒	☐ Clinical data
☒	☐ Dual use research of concern
☒	☐ Plants

Methods

n/a	Involved in the study
☒	☐ ChIP-seq
☒	☐ Flow cytometry
☒	☐ MRI-based neuroimaging

Animals and other research organisms

Policy information about studies involving animals; ARRIVE guidelines recommended for reporting animal research, and Sex and Gender in Research

Laboratory animals	None.
Wild animals	The work was carried out on multiple adult specimen from the following spider species of unknown age: Cheiracanthium punctorium, Meta menardi, Thanatus vulgaris, Larinioides sclopetarius, Psalmopoeus reduncus.
Reporting on sex	Spiders were used for this study at equal sex ratios to exclude sex-biased effects as good as possible.
Field-collected samples	Before sampling, spiders were kept at room temperature with a ~12-h photoperiod for at least 1 week in appropriately-sized plastic enclosures (P. reduncus = 30x30x30 cm, all others 10x10x10 cm) containing potting soil as a substrate.

Ethics oversight	According to the German regulations, work with arthropods does not require ethic approval unless for some selected taxa. None of the herein investigated species falls under these reguations, neither are they protected nor did they were taken from protected areas. hence, no ethics approval was needed.

Note that full information on the approval of the study protocol must also be provided in the manuscript.

Plants

Seed stocks	Not applicable.
Novel plant genotypes	Not applicable.
Authentication	Not applicable.
